# Are primary care factors associated with hospital episodes for adverse drug reactions? A national observational study

**DOI:** 10.1136/bmjopen-2015-008130

**Published:** 2015-12-24

**Authors:** Ailsa J McKay, Roger B Newson, Michael Soljak, Elio Riboli, Josip Car, Azeem Majeed

**Affiliations:** 1 Department of Primary Care and Public Health, Imperial College London, London, UK; 2 School of Public Health, Imperial College London, London, UK; 3 Department of LKCMedicine, Imperial College London—Nanyang Technological University, Singapore, Singapore

**Keywords:** EPIDEMIOLOGY, PRIMARY CARE, PUBLIC HEALTH

## Abstract

**Objective:**

Identification of primary care factors associated with hospital admissions for adverse drug reactions (ADRs).

**Design and setting:**

Cross-sectional analysis of 2010–2012 data from all National Health Service hospitals and 7664 of 8358 general practices in England.

**Method:**

We identified all hospital episodes with an International Classification of Diseases (ICD) 10 code indicative of an ADR, in the 2010–2012 English Hospital Episode Statistics (HES) admissions database. These episodes were linked to contemporary data describing the associated general practice, including general practitioner (GP) and patient demographics, an estimate of overall patient population morbidity, measures of primary care supply, and Quality and Outcomes Framework (QOF) quality scores. Poisson regression models were used to examine associations between primary care factors and ADR-related episode rates.

**Results:**

212 813 ADR-related HES episodes were identified. Rates of episodes were relatively high among the very young, older and female subgroups. In fully adjusted models, the following primary care factors were associated with increased likelihood of episode: higher deprivation scores (population attributable fraction (PAF)=0.084, 95% CI 0.067 to 0.100) and relatively poor glycated haemoglobin (HbA1c) control among patients with diabetes (PAF=0.372; 0.218 to 0.496). The following were associated with reduced episode likelihood: lower GP supply (PAF=−0.016; −0.026 to −0.005), a lower proportion of GPs with UK qualifications (PAF=−0.035; −0.058 to −0.012), lower total QOF achievement rates (PAF=−0.021; −0.042 to 0.000) and relatively poor blood pressure control among patients with diabetes (PAF=−0.144; −0.280 to −0.022).

**Conclusions:**

Various aspects of primary care are associated with ADR-related hospital episodes, including achievement of particular QOF indicators. Further investigation with individual level data would help develop understanding of the associations identified. Interventions in primary care could help reduce the ADR burden. ADRs are candidates for primary care sensitive conditions.

**Strengths and limitations of this study**

- We analysed recent data with national coverage.
- Practice-specific data were available for all predictors.
- The analysis was cross-sectional and at practice-level. We can therefore neither infer that the observed associations are causally linked, nor that they persist at the individual level.
- We were unable to directly adjust for prescribing burden.

## Introduction

Adverse drug reactions (ADRs) have been described as the undesirable and unintended effects of drugs further to their anticipated therapeutic impact, at usual therapeutic doses.^1^ They may be predictable or unpredictable, and acceptable or not.^2^ Occurrence is influenced by local practice,^3^ including prescribing systems,^4^ drug monitoring and associated systems,^5^ ^6^ drug interactions and polypharmacy,^7^ ^8^ and individual patient characteristics.^3^ They are caused by both over-the-counter and prescription medications.^9^ They are a major source of iatrogenic harm, and associated with excess morbidity and mortality.^10^ A 2002 review suggested approximately 7% of UK emergency hospital admissions and 4 in 100 UK hospital bed-days are associated with ADRs.^11^ Unadjusted numbers of ADR-related admissions have been increasing since the late 1990s, with rates of increase exceeding those for hospital admissions per se.^12^ ^13^ Enhanced reporting,^14^ population ageing, increasing comorbidity and polypharmacy^15^ are likely to have contributed to these upward trends. The economic cost of these admissions and some other aspects of ADR management was estimated at £750 million per year in 2006.^16^

A recent meta-analysis^17^ concluded that approximately half of the ADRs identified in secondary care are preventable. However, identifying interventions with consistent positive impact on prescribing errors or ADRs has been difficult.^18–20^ Studies linking both prescribing habits and hospital admissions for particular conditions with primary care provision and performance, nevertheless, indicate that modifiable aspects of primary care influence ADR and hospital admission rates. For example, two recent analyses of primary care data support a negative correlation between prescribing errors, and both, practice list size and designation, as a training versus non-training practice.^3^ ^21^ General practitioner (GP) age, sex, handedness of practice and list size have also been linked to ADR reporting (potentially a proxy for pharmacovigilance more generally),^22^ and list size, GP supply and country of qualification with admission rates, for several other particular conditions.^23–27^ Quality and Outcomes Framework (QOF) performance, on both clinical and service access indicators, has been linked to admission rates for various conditions.^23–27^

To further assess the extent to which ADRs might be influenced by primary care, we have here considered, at practice level, associations between ADR-related admissions and practice demographics, patient factors, measures of primary care supply and performance indicators. We hypothesised that lower ADR admission rates would be associated with higher resourcing and performance measures.

## Methods

### Ethics statement

This was a secondary use of administrative data. The only patient-level data used were Hospital Episode Statistics (HES) data provided by the Health and Social Care Information Centre (HSCIC). The remainder of the data were publicly available practice-level data from the HSCIC (http://www.hscic.gov.uk/home). This is also the case with other published UK analyses that have used HES data.

### Study design, data sources and variables

We performed a cross-sectional analysis of 2010–2012 hospital and primary care data from England.

### Outcome data

The admissions data used to generate our outcome variable were extracted from the 2010 to 2012 English HES Admitted Patient Care data. All episodes of in-hospital care delivered in National Health Service (NHS) hospitals or funded by the NHS are included in this data set. This covers the vast majority of emergency admissions. Accident and emergency attendances without subsequent admission are not included. Each database entry (‘episode’) corresponds to an uninterrupted period of care under a particular hospital consultant. A single inpatient admission in one hospital trust (a HES ‘spell’) can therefore include more than one episode. Duplicate entries (0.026% of total) were excluded. National audit of HES admissions data has shown that 89% of primary diagnoses are valid.^28^

We defined ADR-associated episodes as those with an International Classification of Diseases (ICD) 10 diagnosis term containing the terms, ‘drug-induced’, ‘due to [drug]’, ‘induced by [drug]’, ‘adverse effect of correct drug’, or ‘adverse event of drug’. Those with diagnoses of ‘malignant neuroleptic syndrome’, ‘ototoxic hearing loss’, ‘toxic liver disease’, ‘toxic epidermal necrolysis’, ‘drug phototoxic response’, ‘drug photoallergic response’, ‘post-immunisation arthropathy’, ‘complications following infusion, transfusion and therapeutic injection’ and ‘infection following immunisation’, were also included, as were those with a diagnosis field containing an ‘external cause’ code between Y40 and Y59, which indicate that a drug is the expected cause of a particular diagnosis. Drug-associated poisoning was excluded. An exhaustive list of eligible ICD-10 codes is available as online supplementary file S1. We used the general practice linked to each of the included HES episodes to calculate numbers of ADR-associated episodes per practice.

### Predictor variables: practice demographics and performance measures

Various practice demographic and performance measures were used as predictor variables. Data were obtained from the HSCIC. The following predictors were generated from the 2012 General and Personal Medical Services Data:^29^

- Continuous
  1. Practice list size
  2. GP supply: number of full-time equivalent (FTE) GPs/1000 patients
  3. Per cent of GPs ≥50 years
  4. Per cent of female GPs
  5. Per cent of GPs with non-UK primary medical qualifications
- Binary
  Single-handed/multihanded practice.

The practitioner-related data accounted for all GP providers, salaried/other GPs, GP retainers and GP registrars. Overall 2011–2012 practice QOF performance (per cent of maximum score of 1000 points achieved), and the following 2011–2012 QOF clinical and medication management indicators,^30^ were identified as additional predictors:

1. Clinical indicators (as markers for overall clinical quality of care)

- CHD06: The percentage of patients with coronary heart disease in whom the last blood pressure reading (measured in the preceding 15 months) is 150/90 or less
- CHD08: The percentage of patients with coronary heart disease whose last measured total cholesterol (measured in the preceding 15 months) is 5 mmol/L or less;
- STROKE06: The percentage of patients with a history of transient ischaemic attack (TIA) or stroke in whom the last blood pressure reading (measured in the preceding 15 months) is 150/90 mm Hg or less;
- STROKE08: The percentage of patients with TIA or stroke whose last measured total cholesterol (measured in the preceding 15 months) is 5 mmol/L or less;
- DM17: The percentage of patients with diabetes whose last measured total cholesterol within the preceding 15 months is 5 mmol/L or less;
- DM26: The percentage of patients with diabetes in whom the last International Federation of Clinical Chemistry (IFCC)-glycated haemoglobin (HbA1c) is 59 mmol/mol (equivalent to HbA1c of 7.5% in Diabetes Control and Complications Trial units) or less (or equivalent test/reference range depending on local laboratory) in the preceding 15 months;
- DM30: The percentage of patients with diabetes in whom the last blood pressure is 150/90 or less;
- BP05: The percentage of patients with hypertension in whom the last blood pressure (measured in the preceding 9 months) is 150/90 mm Hg or less.

- 2. Medication management (both binary indicators)
  - MEDICINES12: A medication review is recorded in the notes in the preceding 15 months for all patients being prescribed repeat medicines, standard 80%;
  - RECORDS09: For repeat medicines, an indication for the drug can be identified in the records (for drugs added to repeat prescriptions with effect from 2 April 2004), minimum standard 80%.

These particular clinical indicators were selected as they are important indicators related to common conditions relevant to all practices. They apply disproportionately to older age groups and those with multimorbidity (among whom the targets will be more challenging to meet), and reflect the need for long-term monitoring, which can also be difficult to achieve. The 2010–2011 QOF patient experience indicator data were used, as these indicators were dropped in 2011–2012:

1. PE07: Patient experience of access (1). The percentage of patients who, in the GP Patient Survey, indicate that they were able to obtain a consultation with a GP within two working days. (NB: The GP Patient Survey is a national survey run by an independent survey agency for the NHS. 1.4 million adult patients registered with a GP in England are sampled 4×/year. Almost 2 million responses were received in 2010–2011; response rate=36%.)^31^
2. PE08: Patient experience of access (2). The percentage of patients who, in the appropriate national survey, indicate that they were able to book an appointment with a GP more than two days ahead.

### Predictor variables: patient population sociodemographic and comorbidity data

Covariates included descriptors of practice populations. The age and gender distributions of each practice population (at 2011), and their Index of Multiple Deprivation (IMD) scores (from 2010), were obtained from the HSCIC Indicator Portal.^32^ The following variables were produced with these data:

1. Age group (categorical variable using Office for National Statistics (ONS) age-bands);
2. Sex (male/female binary variable);
3. IMD score (continuous variable).

A summary practice population ethnicity variable was produced using 2011 ONS Census data.^33^ The ethnicity categories were collapsed into a ‘per cent white’ variable (per cent belonging to any of the English/Welsh/Scottish/Northern Irish/British, Irish, Gypsy or Irish Traveller or Other White groups).

As disease burden is associated with rates of admissions, prescribing burden and ADRs,^34^ ^35^ a practice morbidity variable was produced by totalling the numbers of practice QOF disease registrations (2011–2012) for coronary heart disease, heart failure, stroke/TIA, hypertension, atrial fibrillation, diabetes mellitus, chronic obstructive pulmonary disease, asthma, epilepsy, hypothyroidism, cancer, palliative care, schizophrenia, bipolar disorder, other psychoses, depression and dementia, and expressing this as a proportion of list size. Comparison with the Charlson Index has indicated that QOF registration data can reasonably estimate morbidity.^36^

### Exclusions

Practices for which a patient count was not available (n=153), with an incomplete set of predictors (n=538) and/or with a list size <500 (n=3), were excluded from analysis.

### Statistical analysis

For each combination of practice, sex and age group, we computed a count of total ADR-related HES episodes for 2010–2012, and fitted Poisson general estimating equation (GEE) regression models to these data, using Huber variances clustered by practice, with an exposure variable equal to the number of patients in that practice with that gender and age group. For estimating crude rates by gender and age group, we used GEEs with zero correlation. For estimating effects of practice-level predictors, we used GEEs with exchangeable correlation. The parameters of the practice-effects models were a base ADR rate for each combination of gender and age group, and rate ratios corresponding to practice-specific risk factors, which were constant within each practice. For each risk factor, we fitted an unadjusted model, the parameters of which were the base ADR rates and risk ratios for that factor, using binary indicators for binary factors and the quadratic reference-spline method for continuous factors.^37^ We then fitted an adjusted model, containing the base rates and rate ratios for all the risk factors. For each factor (continuous or binary), we estimated the adjusted and unadjusted population attributable fraction (PAF), comparing ADR rates between the real-world scenario and a hypothetical scenario where that factor was at the base level for all participants.^38^ Table 1 displays the baseline and other reference points for all predictors. The reference-spline models used allow the real world to be compared with a hypothetical scenario, in which all practices had the baseline level of a continuous covariate. The PAF is then the proportion of ADRs attributable to living in the real world, instead of in the hypothetical scenario. For instance, in the case of GP supply (FTE/1000 patients), the real world is compared to a hypothetical scenario, in which each practice had 7.5 FTEs per 1000 patients. Analyses were carried out using V.13.1 of Stata statistical software.^39^

**Table 1:** Reference points for predictors

Predictor	Baseline reference point	Additional reference points
Patient population		
IMD	10	10, 25, 40
Ethnicity (% white)	100	50, 90, 100
Practice morbidity index (registrations/1000 patients)	0	0, 500, 750
Practice demographics		
GP supply (FTE/1000 patients)	7.5	4.5, 6, 7.5
Handedness of practice*	0	0, 1
GPs >50 years (%)	0	0, 50, 100
GPs with non-UK qualifications (%)	0	0, 50, 100
Female GPs (%)	0	0, 50, 100
QOF indicator achievement (%)		
Total QOF points	100	90, 95, 100
PE07	100	60, 80, 100
PE08	100	60, 80, 100
CHD06	100	80, 90, 100
CHD08	100	60, 80, 100
STROKE06	100	80, 90, 100
STROKE08	100	60, 80, 100
DM17	100	60, 80, 100
DM26	100	40, 70, 100
DM30	100	80, 90, 100
BP05	100	60, 80, 100
MED12	100	0, 100
RECORD09	100	0, 100

*Multihanded, 0, single-handed, 1.

FTE, full-time equivalent; GP, general practitioner; IMD, Index of Multiple Deprivation; QOF, Quality and Outcomes Framework.

## Results

### Summary statistics

After removal of duplicates, 212 813 ADR-related HES episodes were identified. Following practice exclusions (as above), 7664 (91.7% of 8358) practices remained, with 53 422 119 registered patients. These included practices that were associated with 201 246 (94.6%) of the identified HES episodes; 72.1% of these episodes (n=145 077) were discrete admissions to an NHS Trust (ie, did not occur within the same HES spell). Table 2 displays the number of episodes containing ADR-related ICD-10 codes, by ICD-10 chapter. Most episodes were identified by an ‘external cause’ code, as anticipated in view of the limited number of primary diagnosis codes that attribute a diagnosis to a drug. It is likely that some episodes had both, diagnosis and external cause codes, indicative of an ADR, as the information each provides (disease attributed to drug, and drug considered responsible, respectively), is different. It is also possible that some individuals received more than one ADR diagnosis.

**Table 2:** Distribution of identified ADR-related episodes by ICD-10 chapter

ICD-10 chapter/subdivision (title)	Number of episodes identified	Percentage of episodes identified
ADR-related episodes identified by primary diagnosis code		
III (Diseases of the blood and blood-forming organs and certain disorders involving the immune mechanism)	1047	0.5
IV (Endocrine, nutritional and metabolic diseases)	5899	2.9
V (Mental and behavioural disorders)	156	0.1
VI (Diseases of the nervous system)	7476	3.7
VII (Diseases of the eye and adnexa)		
VIII (Diseases of the ear and mastoid process)		
IX (Diseases of the circulatory system)	10 834	5.4
X (Diseases of the respiratory system)	790	0.4
XI (Diseases of the digestive system)	704	0.3
XII (Diseases of the skin and subcutaneous tissue)	9818	4.9
XIII (Diseases of the musculoskeletal system and connective tissue)	2661	1.3
XIV (Diseases of the genitourinary system)	2285	1.1
XIX (Injury, poisoning and certain other consequences of external causes)	11 390	5.7
Total identified episodes with ADR-related primary diagnosis codes	53 226	26.4
ADR-related episodes identified by external cause code (under Chapter XX: External causes of morbidity and mortality, section Y40-Y59: Drugs, medicaments and biological substances causing adverse effects in therapeutic use)		
Y40: Systemic antibiotics	17 231	8.6
Y41: Other systemic anti-infectives and antiparasitics	3999	2.0
Y42: Hormones and their synthetic substitutes and antagonists not elsewhere classified	16 724	8.3
Y43: Primarily systemic agents	44 703	22.2
Y44: Agents primarily affecting blood constituents	9232	4.6
Y45: Analgesics, antipyretics and anti-inflammatory drugs	23 753	11.8
Y46: Antiepileptics and antiparkinsonism drugs	3910	1.9
Y47: Sedatives, hypnotics and antianxiety drugs	1682	0.8
Y48: Anaesthetics and therapeutic gases	1799	0.9
Y49: Psychotropic drugs not elsewhere classified	6794	3.4
Y50: Central nervous system stimulants not elsewhere classified	201	0.1
Y51: Drugs primarily affecting the autonomic nervous system	8551	4.2
Y52: Agents primarily affecting the cardiovascular system	21 019	10.4
Y53: Agents primarily affecting the gastrointestinal system	2546	1.3
Y54: Agents primarily affecting water-balance and mineral and uric acid metabolism	15 535	7.7
Y55: Agents primarily acting on smooth and skeletal muscles and the respiratory system	1412	0.7
Y56: Topical agents primarily affecting skin and mucous membrane and ophthalmological, otorhinolaryngological and dental drugs	2788	1.4
Y57: Other and unspecified drugs and medicaments	8548	4.2
Y58: Bacterial vaccines	492	0.2
Y59: Other and unspecified vaccines and biological substances	921	0.5
Total identified episodes with ADR-related external cause code	184 442	91.7
Total ADR-related episodes	201 246	

The number of identified Hospital Episode Statistics episodes with ADR-related ICD-10 codes, by ICD-10 chapter and subdivisions of Chapter XX (External causes of morbidity and mortality).

ADR, adverse drug reaction; ICD, International Classification of Diseases.

Practice admission and demographic characteristics, the nature of their patient populations and their QOF performance outcomes, are summarised in table 3. Clustering around high levels of achievement was apparent for many of the QOF outcomes.

**Table 3:** Practice characteristics

	Median	IQR	Per cent
*HES episodes associated with ADRs (total count 2010–2012)*	19	7–38	
Patient population characteristics			
Patient age (% >65 years)	16.0	11.8–19.5	
Patient sex (% female)	50.3	49.0–51.2	
Patient ethnicity (% white)	92.8	76.5–97.2	
Patient morbidity score (registrations/1000 patients)	500.4	424.8–568.8	
IMD	21.7	13.7–32.0	
Practice characteristics			
Practice list size (1000s)	6.2	3.7–9.4	
GP supply (FTE/1000 patients)	6.0	4.9–7.5	
Handedness of practice (% single-handed)			10.2
GPs >50 years (%)	40.0	22.2–60.0	
Female GPs (%)	50.0	33.3–60.0	
GPs with non-UK qualifications (%)	20.0	0.0–50.0	
QOF indicator achievement			
Total QOF points (%)	98.6	96.8–99.4	
PE07 (%)	84.4	76.8–91.1	
PE08 (%)	77.8	66.5–87.5	
CHD06 (%)	90.6	87.7–93.3	
CHD08 (%)	80.2	75.9–84.5	
STROKE06 (%)	89.2	85.5–92.3	
STROKE08 (%)	77.8	72.4–82.5	
DM17 (%)	81.9	77.8–85.6	
DM26 (%)	70.2	65.0–75.1	
DM30 (%)	90.4	87.4–93.2	
BP05 (%)	80.3	76.2–84.0	
MED12 (% of practices achieving target)			96.8
RECORD09 (% of practices achieving target)			93.5

Median and IQR for continuous variables, and percentage of practices single-handed, and achieving QOF indicators MED12 and RECORD09, are displayed.

ADR, adverse drug reaction; FTE, full-time equivalent; GP, general practitioner; HES, Hospital Episode Statistics; IMD, Index of Multiple Deprivation; QOF, Quality and Outcomes Framework.

Table 4 displays ADR-related episode rates by patient age and sex. Relatively high rates were apparent in the very young and older age groups. Post 0–4 years (for whom rates=0.76/1000 person-years, 95% CI 0.70 to 0.81), rates increased with age, from 0.37 (0.34 to 0.40) per 1000 person-years among the 5–14 years age group, to 12.3 (11.9 to 12.6) per 1000 person-years among the ≥85 years age group. Rates were also higher among females compared with males: 2.10 (2.06 to 2.14) vs 1.66 (1.63 to 1.70) per 1000 person-years, respectively.

**Table 4:** ADR-related hospital episode rates by age and sex

	Males			Females			Pooled		
Age group	ADRs (1000s)	Person-years (1000s)	IR (95% CI)	ADRs (1000s)	Person-years (1000s)	IR (95% CI)	ADRs (1000s)	Person-years (1000s)	IR (95% CI)
0–4	2.6	3288.4	0.78 (0.73 to 0.84)	2.3	3131.6	0.73 (0.64 to 0.82)	4.9	6420.0	0.76 (0.70 to 0.81)
5–14	2.5	6122.2	0.41 (0.36 to 0.46)	1.9	5838.0	0.33 (0.29 to 0.37)	4.4	11960.2	0.37 (0.34 to 0.40)
15–44	10.8	22199.5	0.49 (0.47 to 0.50)	16.5	21646.6	0.76 (0.74 to 0.78)	27.3	43846.1	0.62 (0.61 to 0.64)
45–64	22.5	13874.6	1.62 (1.55 to 1.70)	27.4	13494.8	2.03 (1.96 to 2.10)	49.9	27369.3	1.82 (1.77 to 1.88)
65–74	19.8	4394.8	4.51 (4.35 to 4.68)	20.2	4696.2	4.31 (4.19 to 4.43)	40.1	9091.0	4.41 (4.29 to 4.52)
75–84	21.0	2546.6	8.23 (8.00 to 8.46)	25.1	3265.5	7.68 (7.48 to 7.89)	46.0	5812.1	7.92 (7.75 to 8.10)
85+	9.4	768.4	12.24 (11.63 to 12.89)	19.4	1577.2	12.28 (11.95 to 12.61)	28.8	2345.6	12.27 (11.93 to 12.61)
Total	88.5	53194.4	1.66 (1.63 to 1.70)	112.7	53649.8	2.10 (2.06 to 2.14)	201.2	106844.2	1.88 (1.85 to 1.92)

The number of analysed person-years by combination of age group and sex, associated numbers of ADR-related episodes and corresponding incidence rates, is displayed.

ADR, adverse drug reaction; IR, incidence rate.

### ADR episodes and practice characteristics

The regression analysis outcomes are reported as unadjusted and adjusted PAFs (table 5). These describe, for each predictor, the proportional difference in ADR-related episode rates associated with the difference between the reference scenario for that variable (baseline in table 1) and the sample scenario. The unadjusted and adjusted incidence rate ratios associated with each of the reference points for each factor (as per table 1) are reported in online supplementary file S2. In fully adjusted models, the following factors were associated with increased likelihood of ADR-related episode: higher deprivation scores, higher GP supply, a higher proportion of GPs with UK qualifications, higher total QOF achievement rates, lower performance on QOF indicator DM26 (ie, relatively poor HbA1c control among patients with diabetes) and higher performance on indicator DM30 (ie, relatively good blood pressure control among patients with diabetes). Examination of the rate ratios corresponding to HES episode rates in the scenarios where either 50% or 100%—vs 0%—of GPs held non-UK qualifications, however, suggested a non-linear association between ADR-related episodes and country of qualification (adjusted rate ratio (ARR) for 50% vs 0%=0.92 (95% CI 0.88 to 0.97; p=0.0025), whereas ARR for 100% vs 0%=0.97 (0.91 to 1.04; p=0.42); online supplementary file S2). Additionally, the rate ratio corresponding to the episode rates in the scenario where binary QOF indicator RECORD09 was universally not achieved, compared with universally achieved, was indicative of a bottom-end negative association between indicator achievement (drug indications noted in patient records) and episode rates (ARR for indicator non-achievement vs achievement=1.08 (1.00 to 1.16); p=0.046).

**Table 5:** Associations between hospital episodes and primary care factors: population-attributable fractions

	Unadjusted PAF (95% CI)*	p Value	Adjusted PAF (95% CI)†	p Value
Patient population factors				
IMD	**0.089 (0.073 to 0.106)**	**4.9×10^−24^**	**0.084 (0.067 to 0.100)**	**7.3×10^−22^**
Patient ethnicity (% white)	0.009 (−0.009 to 0.027)	0.32	−0.004 (−0.032 to 0.023)	0.78
Practice morbidity index (registrations/1000 patients)	**0.041 (0.024 to** **0.059)**	**5.8×10^−6^**	0.175 (−0.053 to 0.354)	0.12
Practice factors				
GP supply (FTE/1000 patients)	−0.014 (−0.033 to 0.004)	0.13	−**0.016** (−**0.026 to** −**0.005)**	**0.0046**
Handedness of practice	0.001 (−0.002 to 0.003)	0.64	0.001 (−0.002 to 0.005)	0.44
GPs >50 years (%)	−0.041 (−0.089 to 0.005)	0.082	−0.023 (−0.072 to 0.024)	0.35
GPs with non-UK qualifications (%)	−0.007 (−0.029 to 0.014)	0.51	−**0.035** (−**0.058 to** −**0.012)**	**0.0025**
Female GPs (%)	0.022 (−0.032 to 0.073)	0.42	0.049 (−0.024 to 0.117)	0.19
QOF indicator achievement (%)				
Total QOF points	−0.002 (−0.018 to 0.013)	0.78	−**0.021** (−**0.042 to 0.000)**	**0.045**
PE07	0.041 (−0.012 to 0.092)	0.12	0.008 (−0.055 to 0.067)	0.80
PE08	**0.062 (0.003 to 0.118)**	**0.04**	0.031 (−0.039 to 0.096)	0.37
CHD06	−0.006 (−0.094 to 0.074)	0.88	−0.072 (−0.208 to 0.049)	0.26
CHD08	−0.033 (−0.171 to 0.088)	0.61	−0.135 (−0.317 to 0.021)	0.094
STROKE06	0.058 (−0.008 to 0.120)	0.086	0.076 (−0.008 to 0.153)	0.075
STROKE08	0.039 (−0.064 to 0.132)	0.44	0.031 (−0.088 to 0.137)	0.59
DM17	0.125 (−0.007 to 0.240)	0.063	0.128 (−0.023 to 0.257)	0.092
DM26	**0.370 (0.222 to 0.490)**	**1.8×10^−5^**	**0.372 (0.218 to 0.496)**	**3.1×10^−5^**
DM30	−0.031 (−0.120 to 0.050)	0.46	−**0.144** (−**0.280 to** −**0.022)**	**0.02**
BP05	0.073 (−0.086 to 0.208)	0.35	0.138 (−0.069 to 0.304)	0.18
MED12	0.001 (−0.002 to 0.004)	0.55	0.000 (−0.003 to 0.003)	0.98
RECORD09	**0.005 (0.000 to** **0.009)**	**0.048**	0.005 (−0.000 to 0.009)	0.053

Bold typeface denotes p<0.05. Unadjusted and adjusted PAFs associated with each primary care factor are displayed. Each fraction refers to the difference between the baseline scenario in table 1, and the sample scenario.

*Adjusted for practice, patient population age and sex.

†Adjusted for patient population age, sex, ethnicity, morbidity score and IMD, GP age, sex and country of qualification, and practice list size, handedness and QOF achievement on the indicators listed.

FTE, full-time equivalent; GP, general practitioner; IMD, Index of Multiple Deprivation; PAF, population attributable fraction; QOF, Quality and Outcomes Framework.

## Discussion

### Summary of results

We aimed to investigate associations between ADR-related HES episodes and various aspects of primary care, including performance, in an observational study of 2010–2012 data. In our sample, the number of ADR-related episodes, and their distribution by population age and sex, was consistent with previous studies.^12^ Higher deprivation scores, higher GP supply, a higher proportion of GPs with UK qualifications, high total QOF achievement, relatively poor HbA1c control among patients with diabetes, relatively good blood pressure control among patients with diabetes and potentially lower recording of drug indications in patient records, were positively associated with increased likelihood of ADR-related episodes.

### Comparison with the existing literature

The association between ADR-related episodes and country of medical qualification was non-linear, and potentially spurious in a context of multiple comparisons and likely residual confounding. Country of qualification has previously been associated with unplanned cancer admissions, but in that case, non-UK qualification was associated with increased likelihood of admission.^27^ Similar variety in direction of effect on admission rates has been observed for GP supply. Where positive correlations between supply and admissions have been observed (as here, and previously for stroke admissions^25^), this could potentially reflect a loss of continuity of care due to care for individual patients being shared by a larger number of GPs. It is also plausible that more GPs per patient would enhance rates of identification and reporting of ADRs, rather than ADR occurrence. It is difficult to imagine that more GPs would have a negative impact on ADR episode rates per se.

The observed effect of deprivation is in keeping with its consistent positive association with emergency admission rates—both generally, and for various specific conditions, including ADRs.^27^ ^40–42^ Further studies have linked lower socioeconomic status with greater polypharmacy, higher prescription rates for drugs commonly implicated in ADRs and higher drug dosage,^43^ ^44^ with dosage reportedly higher despite adjustment for multimorbidity.

We are cautious about the apparent association between higher total QOF achievement and ADR episodes in view of the small effect size, multiple comparisons and a high degree of clustering around high achievement. High total QOF achievement has previously been associated with a reduced likelihood of admission for both cancer and angina.^26^ ^45^ This is not necessarily out of keeping with our observation, however, as many QOF indicators are directly or indirectly associated with prescribing. That is, prescribing burden may be part of the apparent effect of overall QOF achievement. The observed association between the DM30 blood pressure control indicator and ADR episodes provides an example of a target that may be associated with increased rates of ADRs, via higher prescribing rates. A recent meta-analysis suggested that relatively tight blood pressure control among those with diabetes is associated with higher risk of significant adverse events, although this was with control to lower levels than we have specifically investigated here.^46^ It is also possible that higher QOF achievement is reflective of relatively high-quality care in general, and thus, again, that this is associated with enhanced identification and reporting of ADRs, rather than ADR occurrence per se.

In contrast with blood pressure control, better HbA1c control was observed to be negatively associated with HES episode rates. Potentially relatively high HbA1c reflects treatment resistance, and higher levels of oral hypoglycaemic agent and insulin prescribing, which are known risk factors for ADR-related admissions.^47^ Reverse causality—whereby ADRs could impact on treatment adherence, or the treatment options available, and therefore QOF performance—may also be relevant.

Although we did not observe a significant association between either of the medication management QOF indicators and episodes when considering PAFs, the rate ratios calculated did suggest a small negative association between recording of drug indications and ADR-related episodes. As the record-related data were binary and clustered at high levels, further study with data that provide more information would be of interest.

### Strengths and limitations

Previous studies have considered associations between primary care factors and prescribing errors/high-risk prescribing,^3^ ^21^ but so far as we are aware, this is the first study to investigate associations between primary care factors and ADR-related hospital episodes. The data available covered the majority of the English population, and we were able to control for important covariates.

A limitation of the analysis was its cross-sectional and practice level nature, which means that we can infer neither causal links between the observed associations, nor individual level associations, and the ecological fallacy could operate. Additional limitations include the potential for inaccuracies and inconsistencies in the data sets used. HES data are based on patient notes and therefore reflect the quality of clinical record-keeping. Evidence from several reports suggests ADRs are underestimated in HES data.^12^ ^14^ ^48^ Suggested reasons for under-estimation include under-recognition, under-recording and the limited scope of the relevant ICD-10 codes.^14^ Variation in coding practice by hospital/trust is also possible, but as our sample size was large, this is unlikely to be an important confounder. The data describing GP characteristics and supply did not include locum doctors. As the proportion of primary care delivered by locum doctors is now considerable, discrepancies between the data and practice will exist. This issue also affects the practice handedness variable, which, in view of the contributions made by locum doctors, is likely to represent the management structure of the practice as much as the number of doctors it employs. We were constrained in looking at medical training, as further to those describing ‘non-UK qualification’, data are not easily available for use.

Regarding our definition of ADR-related episodes, we were unable to identify episodes that were unavoidable, due to over-the-counter medications, or to prescribing in secondary care. Moreover, we were unable to identify appropriate high-risk prescribing (ie, instances where the risk of ADR was known and accepted). It is not anticipated that these cases would be systematically associated with particular aspects of primary care in a large data set, but they may have limited the extent to which associations with primary care could be identified. We were also unable to adjust for prescribing burden directly, as we were unable to identify suitable data.

### Implications for research and practice

We have previously suggested that observed associations between primary care factors and admissions for particular conditions support their classification as primary care sensitive conditions (PCSCs).^23^ PCSCs are defined as those conditions for which high-quality primary care can limit disease progression, complications and the need for secondary care.^49^ The concept has arisen in line with the pressures on primary care systems to limit hospital utilisation as demand has increased. However, there remains no widespread consensus on, or empirical basis for, criteria by which to identify PCSCs.^50^ ADRs have not typically been considered PCSCs,^51^ ^52^ but our data indicate that they are likely sensitive to changes in primary care practice. Classifying ADRs as PCSCs could help encourage engagement with the issue, and allocate resources for investigation and implementation of strategies to reduce incidence, at the primary care level. Specific suggestions regarding strategies are difficult to make without further analyses to help understand some of the associations identified.

A particular issue raised by our analysis is the possibility that QOF targets may act to tip relatively high-risk prescribing decisions in favour of prescribing. This suggestion has been made previously,^53^ and previous specific concerns about blood pressure targets have led the National Institute for Health and Care Excellence to apply age-caps to hypertension treatment targets, where evidence suggests treatment benefit is limited to certain age groups.^54^ Further investigation of the associations identified using individual level data, which would allow meaningful comparisons of effect size by age and ethnicity, would help to demonstrate if there are particular subgroups at risk of more harm than benefit in the pursuit of particular QOF targets. Consideration of ADRs subsequent to only specific drugs or drug classes would help to determine those implicated in the associations identified. Together, these pieces of information would help inform prescribing guidance that minimises potential prescribing-related harm.

## Conclusions

ADR-related hospital episodes are associated with various primary care factors, including achievement of particular QOF indicators. Further investigation with individual level data, and analysis of both, population and ADR subgroups, would increase our understanding of these associations. ADRs are candidates for PCSCs.
